# Ant backbone phylogeny resolved by modelling compositional heterogeneity among sites in genomic data

**DOI:** 10.1038/s42003-024-05793-7

**Published:** 2024-01-17

**Authors:** Chenyang Cai

**Affiliations:** State Key Laboratory of Palaeobiology and Stratigraphy, Nanjing Institute of Geology and Palaeontology, Chinese Academy of Sciences, Nanjing, 210008 China

**Keywords:** Taxonomy, Entomology

## Abstract

Ants are the most ubiquitous and ecologically dominant arthropods on Earth, and understanding their phylogeny is crucial for deciphering their character evolution, species diversification, and biogeography. Although recent genomic data have shown promise in clarifying intrafamilial relationships across the tree of ants, inconsistencies between molecular datasets have also emerged. Here I re-examine the most comprehensive published Sanger-sequencing and genome-scale datasets of ants using model comparison methods that model among-site compositional heterogeneity to understand the sources of conflict in phylogenetic studies. My results under the best-fitting model, selected on the basis of Bayesian cross-validation and posterior predictive model checking, identify contentious nodes in ant phylogeny whose resolution is modelling-dependent. I show that the Bayesian infinite mixture CAT model outperforms empirical finite mixture models (C20, C40 and C60) and that, under the best-fitting CAT-GTR **+** G4 model, the enigmatic *Martialis heureka* is sister to all ants except Leptanillinae, rejecting the more popular hypothesis supported under worse-fitting models, that place it as sister to Leptanillinae. These analyses resolve a lasting controversy in ant phylogeny and highlight the significance of model comparison and adequate modelling of among-site compositional heterogeneity in reconstructing the deep phylogeny of insects.

## Abstract (web summary)

This study delves into ant Tree of Life using phylogenomic and model comparison methods on extensive datasets, emphasizing the importance of modelling compositional heterogeneity in understanding deep phylogeny of insects.

## Introduction

Ants are the most ubiquitous and ecologically dominant arthropods in terrestrial ecosystems, largely due to the evolution of eusociality^1,2^. A robust phylogeny of the group is key to understanding ant character evolution, species diversification, and biogeography. Over the past two decades, developments in molecular phylogenetics^1–6^, along with the discovery of exceptional Cretaceous fossils^7–10^ have propelled substantial advances in our understanding of ant evolutionary history.

**Fig. 1:**
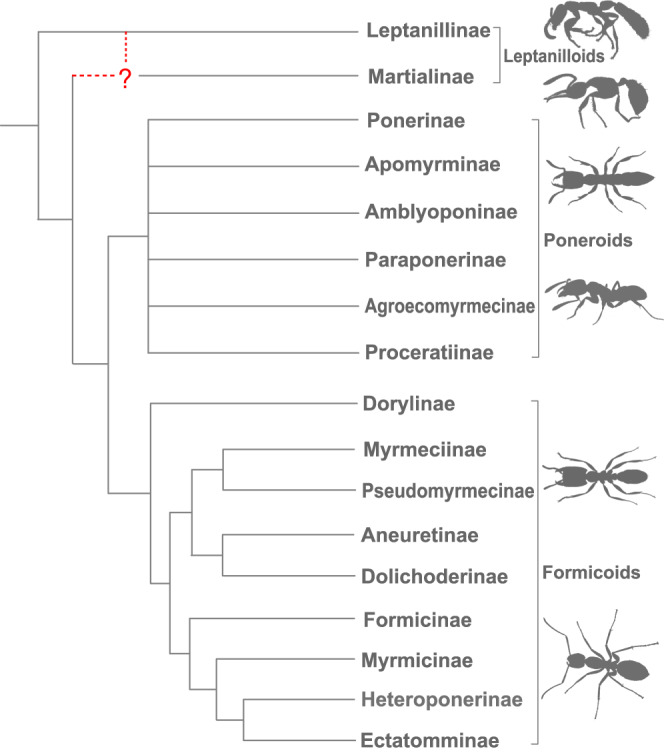
Widely accepted knowledge of phylogenetic relationships among the extant subfamilies of ants. Uncertain deeper relationships focused by the present study are indicated by dashed red lines.

Among the three major groups (formicoids, leptanilloids and poneroids; Fig. 1) of extant ants, the intrafamilial relationships within the formicoids, a clade encompassing the vast majority of ant species, have crystallised from recent molecular phylogenies^2,6,11,12^. By contrast, the intersubfamilial relationships within the poneroid clade are not clear in recent phylogenetic studies, although the monophyly of the clade is strongly supported^2–4^. The most contentious open questions of ant phylogeny lie in the morphologically peculiar leptanilloid clade (Leptanillinae and Martialinae; Fig. 1)^2,4,5^, with some phylogenetic studies rejecting the monophyly of this group^13,14^. The subfamily Martialinae, represented by a sole Neotropical species *Martialis heureka* Rabeling & Verhaagh, was earlier recovered as the sister group to the remainder of the extant ants^13^. However, this hypothesis has been questioned by Kück et al.^14^, who recovered Leptanillinae as a sister group to the extant Formicidae based on improved alignment of the Sanger-sequencing data, alignment masking, and data partitioning. Such inconsistencies may result from confounding factors in phylogenetic analyses such as long-branch attraction^15^ and compositional heterogeneity across lineages^4^. Recently, a genome-scale phylogenomic study based on protein-coding genes and ultra-conserved elements under both concatenation and coalescence methods consistently and strongly supported the monophyly of the leptanilloid clade^2^. Whether *Martialis* or the Leptanillinae are sisters to all other ants is thus the most pressing outstanding question of ant systematics and evolution^1^, since it is fundamental to our understanding of ant phenotype and ecology.

In the phylogenomic era, molecular phylogenies, whether they are maximally supported or not, are often misled by systematic errors when the properties of molecular evolution are not adequately modelled^16–20^. To tackle this issue, I thoroughly explored the recently published Sanger-sequencing^4^ and genome-scale^2^ datasets of ants by testing the fit of substitution models and modelling among-site compositional heterogeneity. My analyses of large-scale datasets under the best-fitting model, selected based on model comparison and posterior predictive model checking, identify modelling-dependent signals and shed light on the contentious early divergences in the tree of ants.

## Results

### Sanger-sequencing datasets of Borowiec et al.^4^

**Fig. 2:**
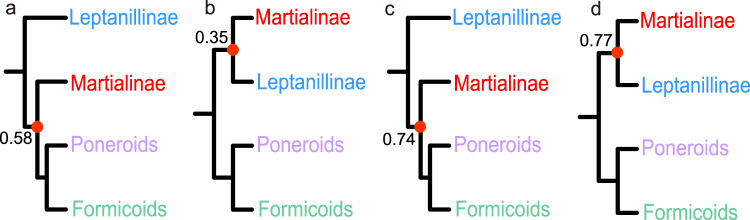
Phylogenetic analyses of Sanger-sequencing datasets from Borowiec et al.^4^ under the site-heterogeneous CAT-GTR + G4 model in PhyloBayes. **a** Full 11-gene matrix. **b** Full matrix with the most AT-rich outgroups excluded. **c** Full matrix with the most GC-rich outgroups excluded. **d** Homogeneous matrix with heterogeneous partitions removed. Weakly supported nodes are labelled in red. The interfamilial relationships among formicoids were well resolved based on all three 7451 NT datasets (**a**–**c**) and consistent with the widely accepted topology. Under the 3995 NT dataset (**d**), the interfamilial relationships of formicoids were not resolved, suggesting a weak phylogenetic signal in the homogeneous matrix. The phylogenetic position of Martialinae was not resolved with confidence in these analyses.

My phylogenetic analyses of three (complete 11-gene matrix and two matrices with the most AT-rich and the most GC-rich outgroups excluded, with 7451 NT sites) of the four datasets presented by Borowiec et al.^4^ under the site-heterogeneous CAT-GTR + G4 model yielded trees (Fig. 2) largely consistent with the ones obtained under the partition model in the original study^4^. The trees based on the complete dataset (Fig. 2a) and the one with the most GC-rich outgroups removed (Fig. 2c) were identical to each other in terms of the ingroup topology and support. Leptanillinae were recovered as sister to all other ants, and *Martialis* was weakly supported as the second-branching lineages (Bayesian posterior probability [BPP] = 0.58 and 0.74, respectively). For the matrix with the most AT-rich outgroups removed (Fig. 2b), *Martialis* was very weakly supported as a sister to Leptanillinae (BPP = 0.35), but all other relationships were congruent with the complete dataset. Due to site filtering, that reduced the phylogenetic signal and compositional bias^4^, the fourth compositionally homogeneous matrix (3995 NT sites) under CAT-GTR + G4 yielded an overall weakly supported tree (Fig. 2d): *Martialis* was weakly supported as sister to Leptanillinae (BPP = 0.77), and the intersubfamilial relationships within the poneroid and formicoid clades too were weakly supported. Overall, the deep relationships among ants, i.e., the systematic placements of Leptanillinae and *Martialis*, were not resolved with confidence based on these four datasets.

### Nuclear genomic datasets of Romiguier et al.^2^

**Fig. 3:**
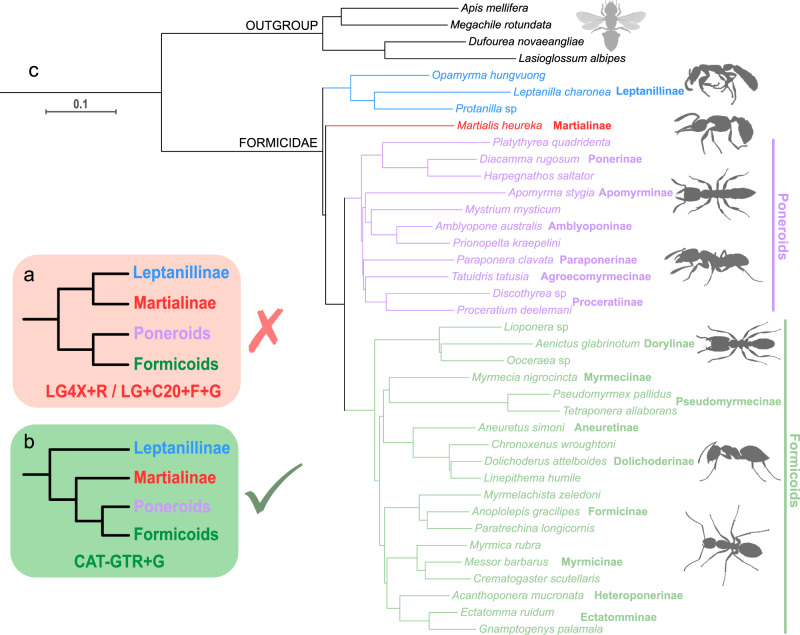
Phylogenomic analyses of filtered nuclear genomic 4151-gene dataset (Matrix 1) from Romiguier et al.^2^. **a** Simplified rejected topology under the LG4X + R and LG + C20 + F + G models in IQ-TREE, showing the relationships of the four lineages of ants. **b** Simplified topology of my preferred tree (**c**). **c** Preferred topology under the site-heterogeneous CAT-GTR + G4 model in PhyloBayes; all nodes are maximally supported (BPP = 1). The outgroup bee silhouette is from Phylopic.

**Fig. 4:**
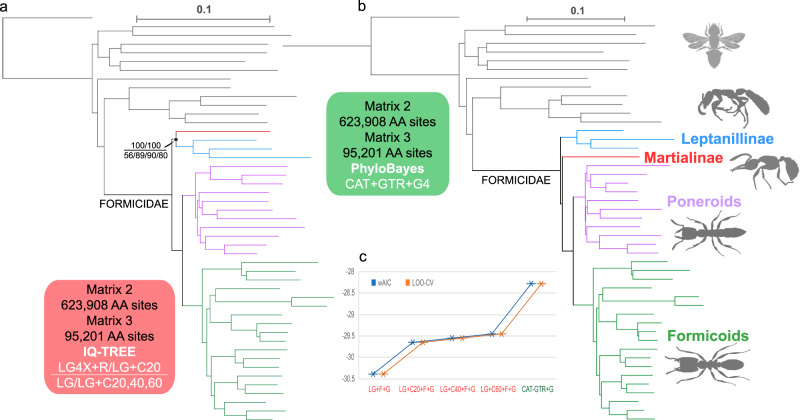
Phylogenomic analyses of filtered nuclear genomic 2343-gene dataset (Matrices 2 and 3) from Romiguier et al.^2^ and model comparison. **a** Topology under the LG4X + R and LG + C20 + F + G models for Matrix 2 and under the LG + F + G, LG + C20 + F + G, LG + C40 + F + G, and LG + C60 + F + G models for Matrix 3 in IQ-TREE; support values for Martialis (red) + Leptanillinae (blue) are shown. **b** Topology inferred from both matrices under the site-heterogeneous CAT-GTR + G model in PhyloBayes; all nodes are maximally supported (BPP = 1). **c** Model comparison based on Matrix 3 shows that CAT-GTR + G4 is clearly better fitting than the other tested four models, including LG + C20 + F + G used in Romiguier et al.^2^. The values displayed on the *y*-axis represent the debiased scores. Abbreviations: LOO-CV leave-one-out cross-validation, wAIC widely applicable information criterion.

In my maximum likelihood analyses using IQ-TREE, Matrix 1, Matrix 2 and Matrix 4 under LG4X + R and LG + C20 + F + G models yielded consistent results (Figs. 3a and 4a; Supplementary Figs. 1, 2 and 4): the clade *Martialis* + Leptanillinae was maximally supported (maximum likelihood bootstrap [MLB] = 100; except for Matrix 4 under the LG4X + R model, MLB = 92), as was the monophyly of the poneroid and formicoid clades (MLB = 100). Similarly, Matrix 3 under the site-homogeneous LG + F + G model and the empirical site-heterogeneous LG + C20, C40 and C60 models resulted in a similar topology to that derived from Matrix 2, although the support values for *Martialis* + Leptanillinae were low (MLB = 56, 89, 90 and 80, respectively; Supplementary Fig. 3). Interestingly, Matrix 5 under LG4X + R, LG + C20 + F + G and GHOST (LG + FO*H4) models consistently yielded different topologies in terms of the placement of *Martialis*: *Martialis* was supported as sister to poneroids + formicoids (MLB = 81, 97 and 73, respectively; Supplementary Figs. 5 and 6).

By contrast, under the site-heterogeneous CAT-GTR + G4 mixture model, all datasets (Matrices 1–5) yielded a consistent topology in terms of the systematic positions of *Martialis* and Leptanillinae (Figs. 3b, c and 4b; Supplementary Figs. 1c, 2c, 3f, 4c and 5c). All nodes in all Bayesian analyses of Matrices 1, 2 and 4 were maximally supported (BPP = 1), suggesting that the phylogenetic signal was strong in these matrices. Leptanillinae was a sister group to all other subfamilies of ants, and *Martialis* was recovered as a sister to the monophyletic poneroids and formicoids. The intersubfamilial relationships of formicoids were identical in all analyses, agreeing with the currently accepted topology based on recent phylogenetic studies^1–4^. The interrelationships of poneroid subfamilies were slightly different between the 4151-gene Matrix 1 and the 2343-gene Matrices 2 and 3. Based on Matrix 1, Ponerinae were maximally supported as sister to the remaining five subfamilies (Fig. 3a; Supplementary Fig. 1c). Based on Matrices 2 and 3, however, Apomyrminae + Amblyoponinae was sister to other lineages (Fig. 4b; Supplementary Figs. 2c and 3f).

### Model comparison

The LOO-CV (leave-one-out cross-validation) and the wAIC (widely applicable information criterion) scores were obtained based on Matrix 3, considering the huge computational burden. The scores were close to each other, suggesting that wAIC is a close approximation of LOO-CV for the 2343-gene amino acid dataset. ∆CV and ∆wAIC were calculated as the difference in the estimated predictive performance between the best-fitting model and another model under consideration. As shown in Fig. 4c, the CAT-GTR + G4 mixture model fitted the dataset better than any of the other models, including the site-homogeneous LG + F + G model, and the LG + C20 + F + G, LG + C40 + F + G, and LG + C60 + F + G models on the amino acid dataset, both according to LOO-CV (∆CV = −28.28 + 29.44 = 1.16) and according to wAIC (∆wAIC = −28.28 + 29.44 = 1.16). The LG + C20 + F + G model used by Romiguier et al.^2^ was clearly better fitting than the LG + F + G model (∆CV = −29.66 + 30.39 = 0.73), but less well fitting than the CAT-GTR + G4 model. Therefore, topologies reconstructed with the CAT-GTR + G4 model were used as the preferred trees for elucidating the relationships of ant subfamilies.

### Posterior predictive model checking

To test whether available models could adequately describe among-site amino acid preferences, I used analyses of site-specific amino acid diversity (PPA-DIV), or the mean number of distinct amino acids observed at each site, based on Matrix 3, as shown in Table 1. The *Z* score was adopted here since the MCMC estimate of the *p*-value is 0. PPA-DIV has a broad and distinctive distribution of *Z* scores across the different tested models (from 156.891 under LG + F + G to 3.974 under CAT-GTR + G4). For the tested dataset, absolute *Z* scores greater than 5 were obtained under the LG + F + G and LG + C20, C40 and C60 models, indicating a strong rejection of the null hypothesis that the model adequately describes the data. PPAs indicated that CAT-GTR + G4 (*Z*-score = 3.974), the model that was favoured based on my model comparison test, describes site-specific amino acid preferences substantially better than other tested models. This result was not surprising, since CAT-GTR + G4 is known to be by far the best-fitting model that can explicitly accommodate among-site compositional heterogeneity^21–24^.

**Table 1:** Comparing model adequacy.

Model	LG + F + G	LG + C20 + F + G	LG + C40 + F + G	LG + C60 + F + G	CAT-GTR + G4
Observed diversity	3.498	3.498	3.498	3.498	3.498
Mean diversity	4.360	3.780	3.768	3.710	3.522
*Z* score	156.891	58.779	48.563	47.983	3.974
*p* value	0	0	0	0	0

Alignments simulated under the CAT-GTR + G4 model have, on average 3.522 amino-acids per site, which is distinctly closer to what is seen on the empirical sequence alignment (3.498 amino-acids per site).

*Z* values of the posterior predictive analyses demonstrate how well each model describes site-specific amino acid preferences.

## Discussion

### Impacts of outgroup choice and data filtering on tree topology

My detailed analyses of both Sanger-sequencing and genome-scale datasets provide a basis for reassessing the subfamilial relationships of ants. As found in the recent phylogenomic study^2^, my results based on multiple supermatrices (variants of the less-outgroup 4151-gene and more-outgroup 2343-gene datasets) demonstrate that outgroup selection does not affect the internal phylogeny of ants when the phylogenetic signal is strong. By contrast, outgroup choice does influence the basal relationships when the much smaller Sanger-sequencing nucleotide datasets are analysed^4^ and this may stem from sequence divergence and a lack of sufficient phylogenetic signal. My phylogenomic analyses also show that data filtering of various degrees and taxon subsampling do not affect the topology of the basal ant phylogeny, when the large-scale matrices are used. Moreover, my comparative phylogenomic results under both site-homogenous and site-heterogeneous models, integrated with formal model comparison, clearly demonstrate that modelling among-site compositional heterogeneity is the key to a natural ant tree of life.

### Significance of model comparison and modelling compositional heterogeneity

Modelling of amino acid replacement is central to phylogenomic inference, particularly so when dealing with deeper relationships and rapid radiations. As such, model comparison is a crucial yet computationally challenging step in modern phylogenomics. In the most recent phylogenomic study of ants^2^, the empirical finite mixture model LG + C20 + F + G was selected to mitigate long-branch-attraction artefacts by modelling among-site compositional heterogeneity. The selection of this particular model (instead of LG + C40 + F + G, LG + C60 + F + G, or other theoretically better-fitting models) was apparently a compromise since runs of supermatrices under the C40 and C60 models (in IQ-TREE) are computationally expensive in terms of both running time and memory requirements. A recent study focusing on model comparison under Bayesian cross-validation, however, shows that amino acid mixture models (CAT models) outperform all single-matrix models (LG, WAG) and free finite mixtures (CAT-GTR + G4) consistently outperform empirical finite mixtures (e.g., LG + C20, C40 and C60)^25^. Not surprisingly, my cross-validation analyses based on the ant dataset reached the same conclusion that CAT-GTR + G4 outperforms other tested models (including LG + C20 + F + G and LG + C60 + F + G). In addition, similarly to a recent simulation study on the rooting of the animal tree^26^, my posterior predictive analyses demonstrate that CAT-GTR + G4 can best describe site-specific amino acid preferences in ant phylogenomics, so CAT should be preferred to C60. Overall, my analyses of genome-scale data highlight the significance of model comparison and adequate modelling of among-site compositional heterogeneity in deciphering the deep phylogeny of ants.

My reanalyses of the 11-loci datasets from Borowiec et al. ^4^ suggest that deeper phylogeny of ants and the position of Martialinae cannot be unambiguously resolved when the phylogenetic signal is weak. Among all of my analyses, the highest support regarding the placement of *Martialis* was 0.77, under the compositionally homogeneous matrix (Fig. 1d). However, in this particular analysis, support for the nodes within formicoids were exceptionally weak, and more importantly, the relationships within formicoids were inconsistent with the widely accepted topology^1,2^ and my phylogenomic results.

### Position of *Martialis* in the ant Tree of Life

The discovery of *Martialis heureka* (Martialinae) based on a single stray worker from the Amazon (north of Manaus, Brazil)^13^ is exciting and perplexing. Since *M. heureka* displays a bizarre combination of both pleisiomorphic and autapomorphic traits, it was placed into its own subfamily. Its precise phylogenetic position has been contentious since its discovery. Rabeling et al. ^13^ recovered *M. heureka* as the sister to all remaining extant subfamilies, while a reanalysis by Kück et al. ^14^ recovered it as the second-branching lineage after Leptanillinae. Subsequent integrated analyses based on a handful of loci^4,27^ continued to reach divergent conclusions. Based a broad sampling and genome sequencing, Romiguier et al.^2^. retrieved high support for the leptanillomorph clade (Leptanillinae and Martialinae) as the sister group to all other extant ants. My reanalyses of multiple datasets from the most comprehensive studies^2,4^ under better-fitting models show that *M. heureka* is sister to all ants except Leptanillinae, agreeing with the conclusion of Kück et al.^14^ but rejecting other topologies^2,4,10,13^. My analyses resolve a lasting enigma in ant phylogeny and offer a backbone topology for investigations of character evolution, biogeography, and ecology of early ants. For instance, Boudinot et al.^10^ recently have adduced some potential synapomorphies of Leptanillomorpha (*Martialis* + Leptanillinae), but the present phylogenomic results suggest that these morphological similarities could be a consequence of convergent acquisition of features adapting these ants to a subterranean lifestyle.

The relationships of formicoid subfamilies have long been concerted^1^, but the intersubfamilial relationships of poneroids remain unsettled. Monophyly of the morphologically heterogeneous poneroids has been recently consistently recovered in molecular phylogenetic studies, including Moreau et al.^6^, some analyses of Brady et al.^11^, Ward and Fisher^28^, Borowiec et al.^4^ and phylogenomic studies^2,5^. In more recent studies^4,5^, many deeper nodes within poneroids were weakly to moderately supported. The resolution of poneroid relationships has been much improved in Romiguier’s et al.^2^ genome-based phylogenomic study. The remaining incongruences in poneroid relationships between the aforementioned study and the present analyses of the filtered 4151-gene dataset (Matrix 1) under CAT-GTR + G4 remain to be addressed by future studies with a broader taxon sampling. Ponerinae are a sister group to the clade (Amblyoponinae, Apomyrminae) + (Paraponerinae, (Agroecomyrmecinae, Proceratiinae)) (Fig. 2a). My phylogenomic study will provide a foundation for understanding ant evolution and comparative studies of evolutionary innovations among ants.

## Methods

### Dataset collation

I used the most comprehensive Sanger-sequencing and nuclear genome alignments from Borowiec et al.^4^ and Romiguier et al.^2^, respectively. The datasets (Sanger-sequencing datasets^29^ and genome-scale datasets^30^) were downloaded from the Zenodo data repository.

For the Sanger-sequencing (11 nuclear loci) data, I used all four nucleotide [NT] matrices generated in Borowiec et al.:^4^ (1) Full 11-locus matrix (123 taxa, 7451 NT sites); (2) Full matrix with the most AT-rich outgroups excluded (117 taxa, 7451 NT sites); (3) Full matrix with the most GC-rich outgroups excluded (117 taxa, 7451 NT sites); and (4) Homogeneous matrix with heterogeneous partitions removed (123 taxa, 3995 NT sites).

For the nuclear genomic data, I used the two BUSCO-gene amino acid [AA] supermatrices from Romiguier et al.:^2^ (1) Fewer-outgroup AA dataset (83 taxa, 4151 single-copy protein-coding genes, 1,692,050 AA sites); and (2) More-outgroup amino acid dataset (188 taxa, 2343 single-copy protein-coding genes, 983,951 AA sites), which was designed to test the impact of outgroup selection on tree inference. To balance taxon sampling of subfamilies, focus on the deeper phylogeny of ants, and, more importantly, speed up computationally heavy Bayesian runs, I subsampled AA sites of the 4151-gene supermatrix and filtered constant sites (to speed up analyses) using BMGE v.1.1^31^, resulting in Matrix 1 (38 taxa, 647,114 AA sites). Similarly, I randomly pruned the outgroup taxa and selected all representative ingroup genera of the 2343-gene supermatrix, and filtered ambiguously aligned sites using BMGE with default setting (-m BLOSUM62, -h 0.5), yielding Matrix 2 (47 taxa, 623,908 AA sites). Additionally, as sensitivity tests of the potential impact of my data filtering and subsampling methods on tree inference, I (1) filtered the 2343-gene supermatrix using a stringent setting (-m BLOSUM30, -h 0.1:0.5) to select slow-evolving AA sites, resulting in Matrix 3 (47 taxa, 95,201 AA sites); (2) removed remotely related outgroup and selected representative ant genera, but kept all AA sites of the 4151-gene supermatrix, resulting in Matrix 4 (17 taxa, 1,692,050 AA sites); and (3) filtered the 4151-gene supermatrix using BMGE with a very stringent setting (-m BLOSUM30 -h 0.2:0.3), resulting in Matrix 5 (82 taxa, 21,902 AA sites).

### Phylogenetic analyses

Phylogenomic analyses of the nuclear genomic datasets, Matrices 1–5, were conducted using the simpler LG4X + R model^32,33^ and the site-heterogeneous model (LG + C20 + F + G)^34^ with IQ-TREE v.2.1.3^35^. For the site-heterogeneous LG + C20 + F + G model, the posterior mean site frequency (PMSF) model^36^ was applied using the respective LG4X + R tree as the guide tree. In addition, the comparatively small Matrix 3 was analysed using the site-homogeneous model (LG + F + G) and the site-heterogeneous models (LG + C40 + F + G, and LG + C60 + F + G) with IQ-TREE v.2.1.3, corresponding to the models used on the following model comparison (see below). For Matrix 5, the heterotachous model (General Heterogeneous evolution On a Single Topology, GHOST^37^) was also tested using IQ-TREE v.2.1.3.

As the subfamilial interrelationships of ants are expected to be affected by long-branch attraction artefacts^1,2^, I used the compositionally site-heterogeneous infinite mixture model CAT-GTR + G4 implemented in PhyloBayes MPI 1.9^38^, which has been proven to be effective for mitigating such a systematic error by modelling across-site compositional heterogeneity. Four Sanger-sequencing matrices (as nucleotides) and five genome-based supermatrices (as amino acids) were analysed under the CAT-GTR + G4 model. For each analysis, two Markov chain Monte Carlo chains were run, and convergence was assessed using the *bpcomp* and *tracecomp* tools implemented in PhyloBayes^39^. Approximately 30% of samples were discarded as burn-in. Detailed information about the PhyloBayes runs (burnin samples, total number of cycles, bpcomp maxdiff and tracecomp minimal overall effective size) is given in the figure caption of each analysis.

### Model comparison

For the filtered genome-scale AA dataset (Matrix 3), I used the comparatively efficient and reliable approaches, i.e., the leave-one-out cross-validation (LOO-CV) and the widely applicable information criterion (wAIC)^40^, to estimate the relative fit of alternative models (CAT-GTR, LG + G, LG + C20, LG + C40, and LG + C60) in the latest PhyloBayes MPI 1.9. The general idea of cross-validation is to split the data set into two subsets, using one subset for training the model and then evaluating the fit of the model over the remaining subset. In the context of Bayesian inference, a natural procedure to implement CV is to average the validation likelihood over the training posterior distribution. The resulting CV score is then log-transformed and averaged over multiple random splits of the original data set into training and validation sets. In leave-one-out CV, each observation is taken in turn and set aside for validation, using the *n* − 1 remaining observations to train the model^40^. The LOO-CV and wAIC scores were compared to determine and select the best-fitting model, based on which my preferred tree of ants was selected.

### Testing model adequacy

Posterior predictive analyses (PPA)^39^ were performed on Matrix 3 using PhyloBayes MPI 1.9 to test whether LG + F + G, LG + C20 + F + G, LG + C40 + F + G, LG + C60 + F + G, or CAT-GTR + G can adequately describe site-specific amino acid preferences for the dataset. These models (especially LG + F + G and LG + C20 + F + G) were selected and tested because they had previously been used in the recent study of ant phylogenomics^2^ that yielded a contradictory topology to my preferred tree.

### Statistics and reproducibility

For Bayesian phylogenetic analyses, I followed the practice as indicated in the manual of PhyloBayes^39,40^, which integrates detailed methods (*bpcomp* and *tracecomp*) for statistical evaluations. Detailed information on the PhyloBayes runs (burnin samples, total number of cycles, bpcomp maxdiff and tracecomp minimal overall effective size) is given in the supplemental figure caption of each analysis. In addition, as sensitivity tests of the potential impact of my data filtering and subsampling methods on tree inference, I further designed three additional supermatrices for comparison.

### Reporting summary

Further information on research design is available in the Nature Portfolio Reporting Summary linked to this article.

## Supplementary information

Supplimental figure 1-6

Reporting Summary

## Data Availability

The data sets and output files generated in our phylogenomic analyses have been deposited in the Dryad Digital Repository [10.5061/dryad.pk0p2ngsj]^41^.
