# Supplementary material for: Ant backbone phylogeny resolved by modelling compositional heterogeneity among sites in genomic data
Source: Commun Biol. 2024 Jan 17;7:106. doi: 10.1038/s42003-024-05793-7 (PMC10794244; doi:10.1038/s42003-024-05793-7)
Supplement: Supplementary file 1 — Supplimental figure 1-6 [file 42003_2024_5793_MOESM1_ESM.pdf]

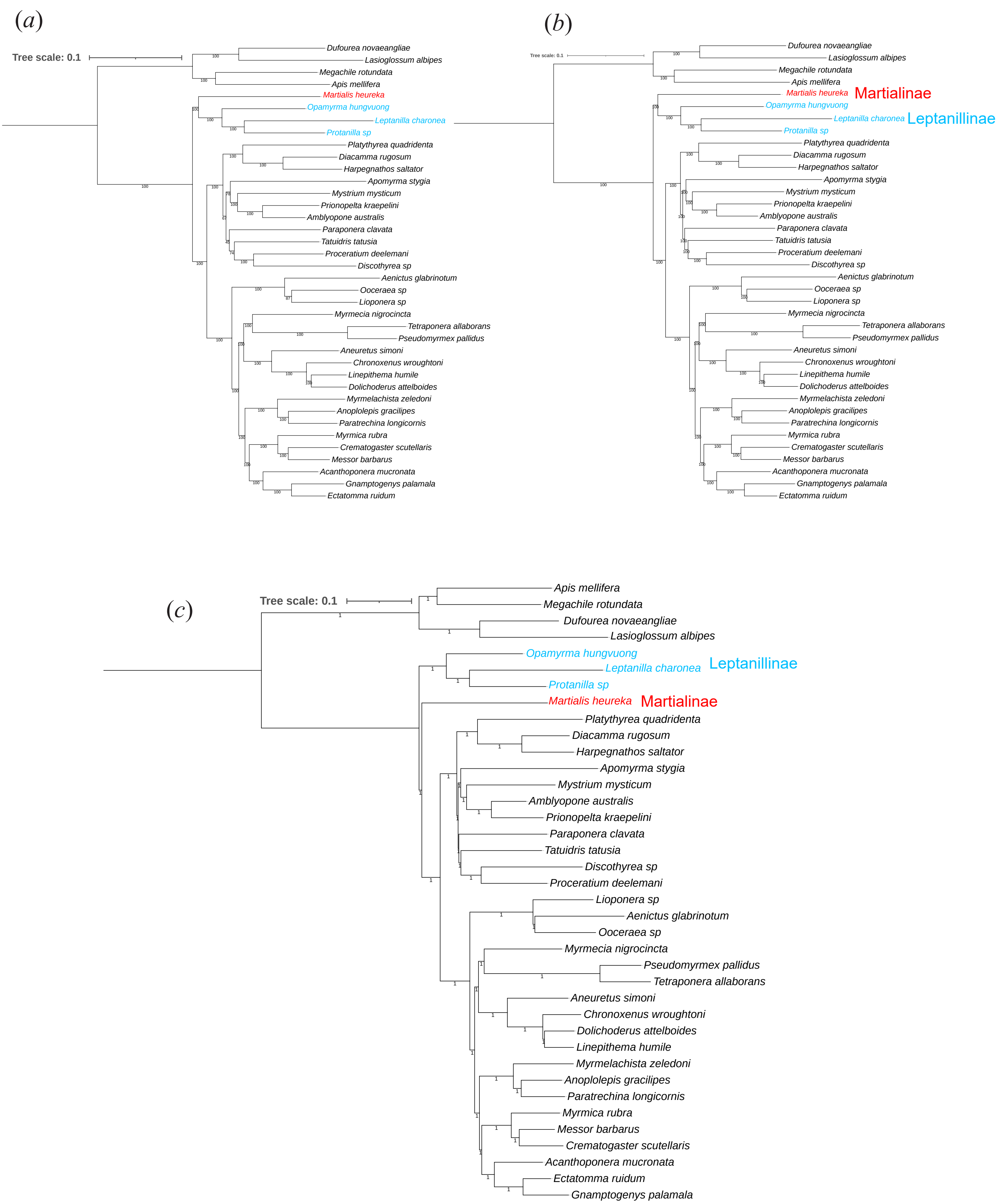

Figure S1. Phylogenomic analyses of filtered nuclear genomic 4,151-gene dataset (Matrix 1) from Romiguier et al. (2022). (*a*) Under the LG4X+R model in IQ-TREE. (*b*) Under the LG+C20+F+G model in IQ-TREE (PMSF, LG4X+R tree as guide tree). (*c*) Preferred topology under the site-heterogeneous CAT-GTR+G4 model in PhyloBayes: Burnin = 470 samples; Total number of cycles = 1424; Bpcomp maxdiff = 0; Trace-comp minimal overall effsize = 52.

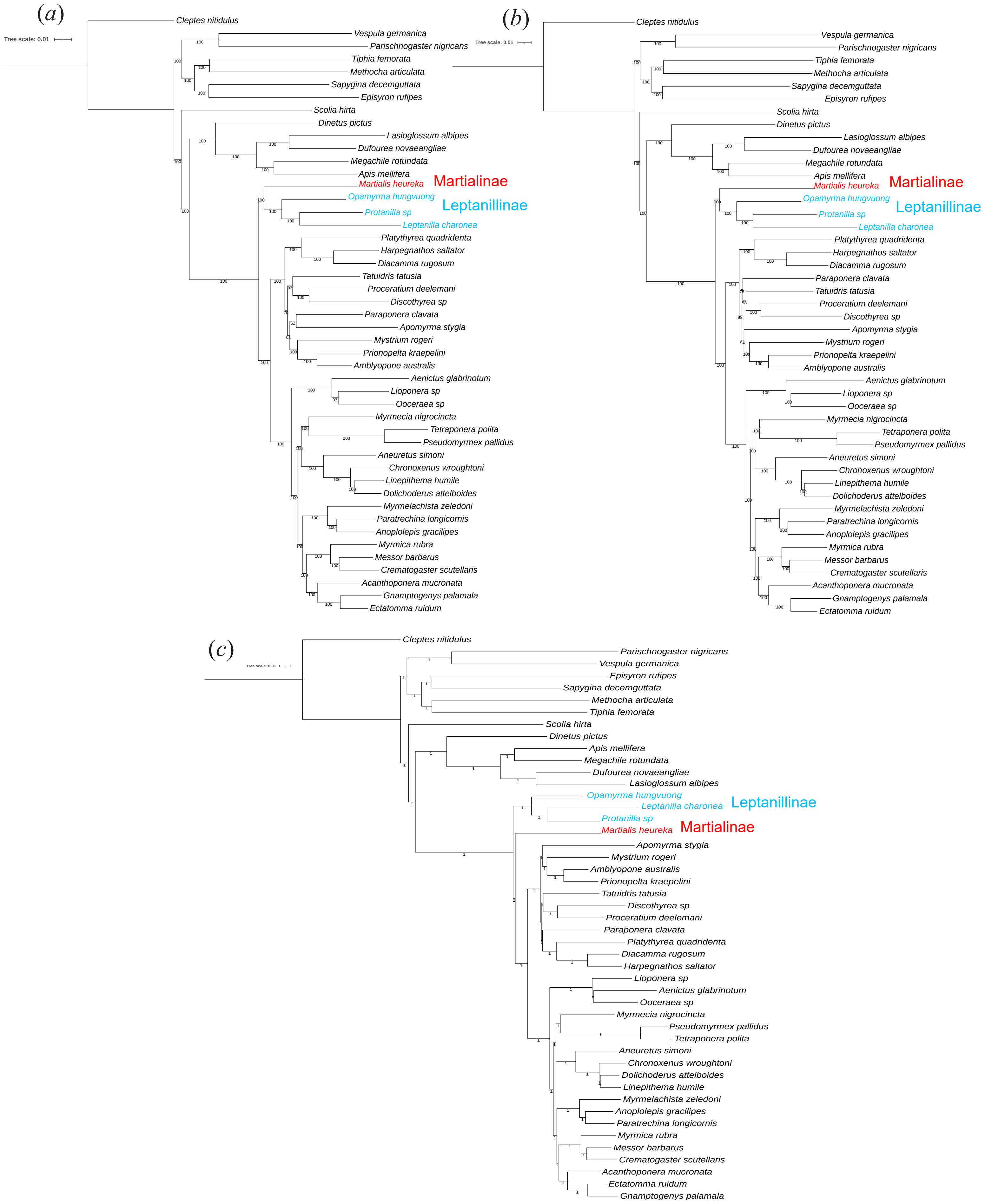

Figure S2. Phylogenomic analyses of filtered nuclear genomic 2,343-gene dataset (Matrix 2) from Romiguier et al. (2022). (*a*) Under the LG4X+R model in IQ-TREE. (*b*) Under the LG+C20+F+G model in IQ-TREE (PMSF, LG4X+R tree as guide tree). (*c*) Preferred topology under the site-heterogeneous CAT-GTR+G4 model in PhyloBayes: Bpcomp maxdiff/meandiff = 0.

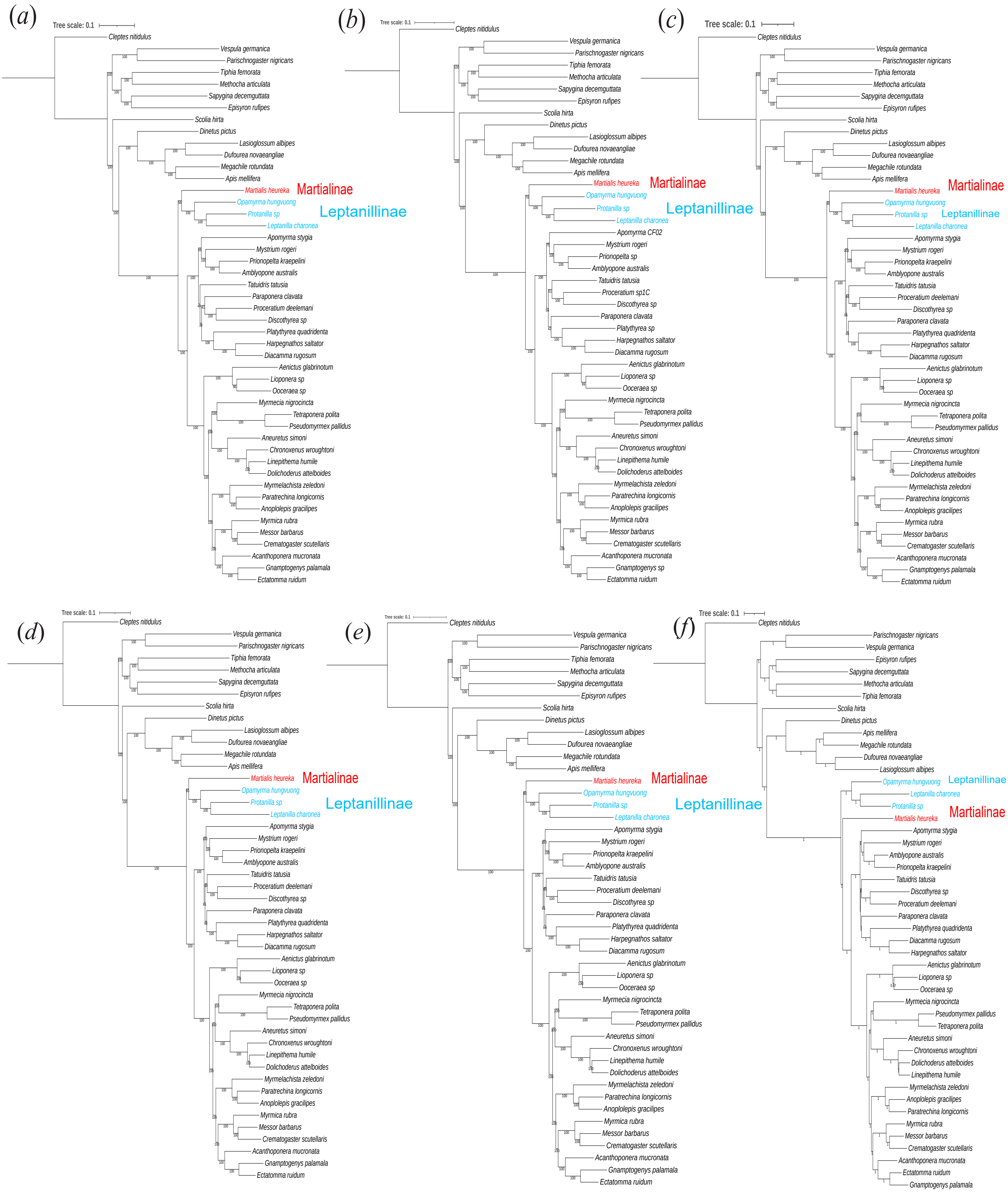

Figure S3. Phylogenomic analyses of filtered nuclear genomic 2,343-gene dataset (Matrix 3) from Romiguier et al. (2022). (*a*) Under the LG+F+G model. (*b*) under the LG4X+R model. (*c*) Under the LG+C20+F+G in IQ-TREE (PMSF, LG4X+R tree as guide tree). (*d*) Under the LG+C40+F+G in IQ-TREE (PMSF, LG4X+R tree as guide tree). (*e*) Under the LG+C60+F+G in IQ-TREE (PMSF, LG4X+R tree as guide tree). (*f*) Preferred topology under the site-heterogeneous CAT-GTR+G4 model in PhyloBayes: Burnin = 2400 samples; Total number of cycles = 7699; Bpcomp maxdiff = 0.1458; Tracecomp minimal overall effsize = 197.

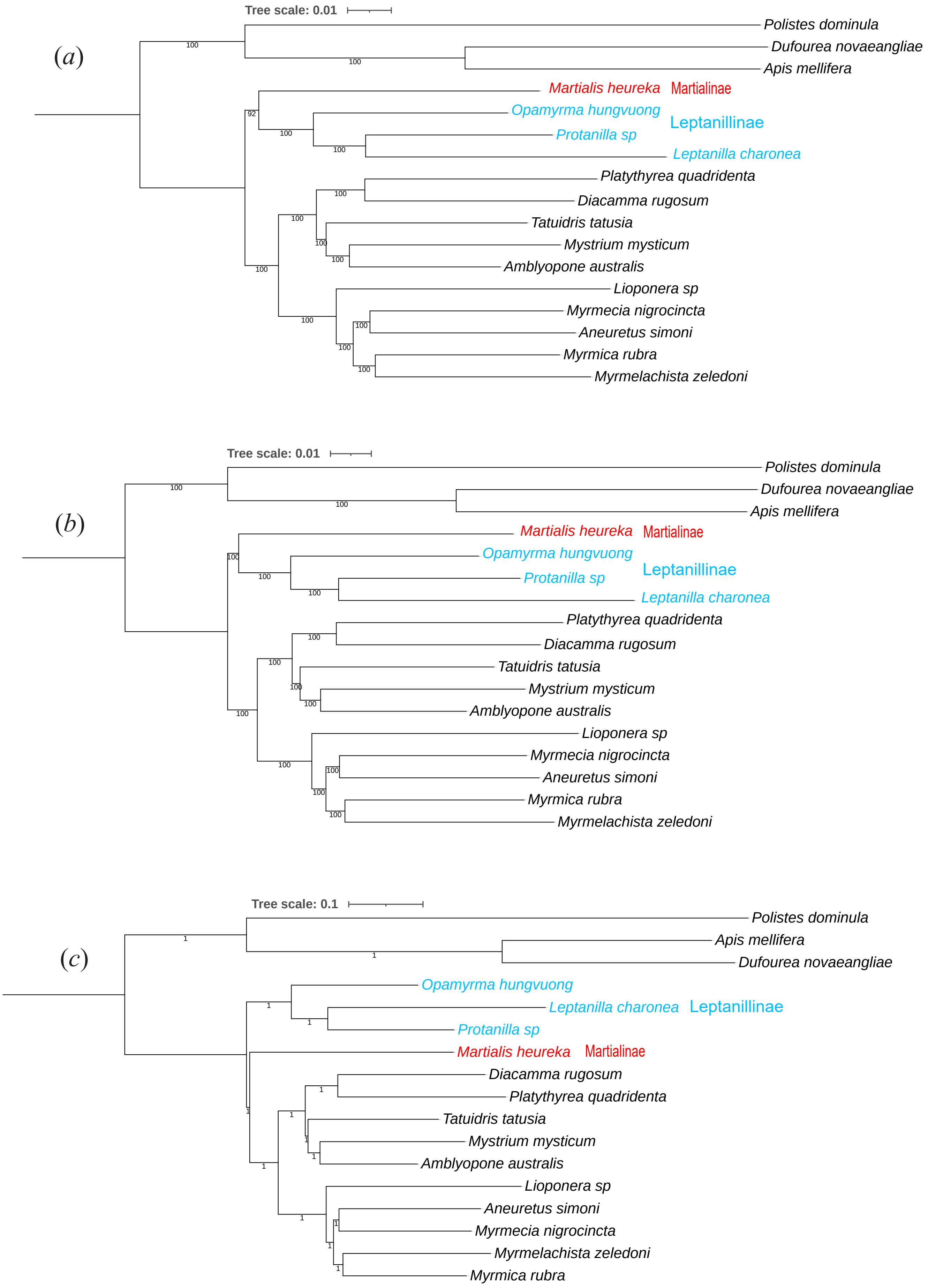

Figure S4. Phylogenomic analyses of subsampled (alignments not trimmed) nuclear genomic 4,151-gene dataset (Matrix 4) from Romiguier et al. (2022). (*a*) Under the LG4X+R model in IQ-TREE. (*b*) Under the LG+C20+F+G model in IQ-TREE (PMSF, LG4X+R tree as guide tree). (*c*) Preferred topology under the site-heterogeneous CAT-GTR+G4 model in PhyloBayes: Burnin = 350 samples; Total number of cycles = 1055; Bpcomp maxdiff = 0; Tracecomp minimal overall effsize = 54.

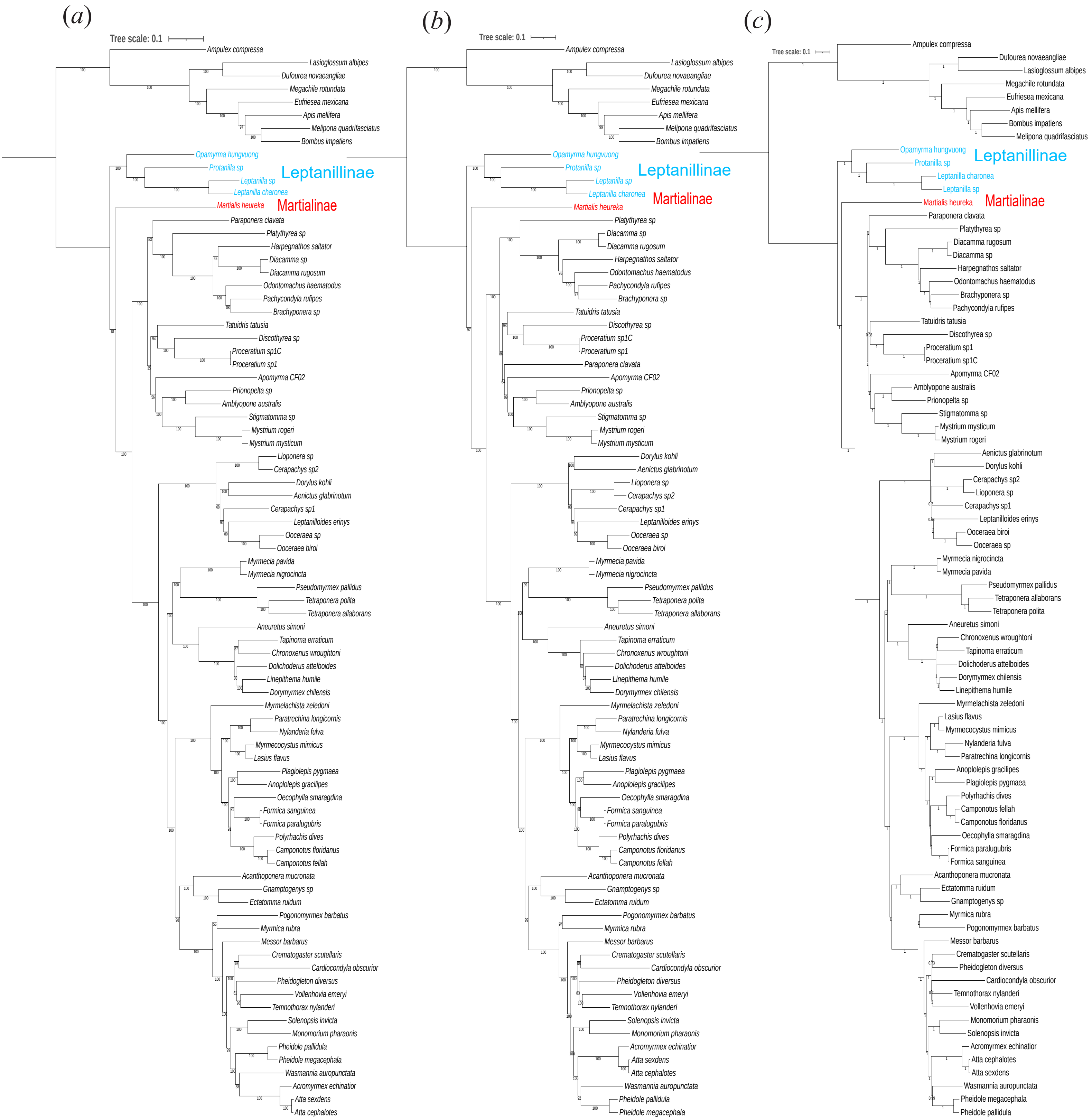

Figure S5. Phylogenomic analyses of trimmed nuclear genomic 4,151-gene dataset (Matrix 5) from Romiguier et al. (2022). (*a*) Under the LG4X+R model in IQ-TREE. (*b*) Under the LG+C20+F+G model in IQ-TREE (PMSF model not used). (*c*) Under the site-heterogeneous CAT-GTR+G4 model in PhyloBayes: Burnin = 600 samples; Total number of cycles = 2065; Bpcomp maxdiff = 0.17; Tracecomp minimal overall effsize = 250.

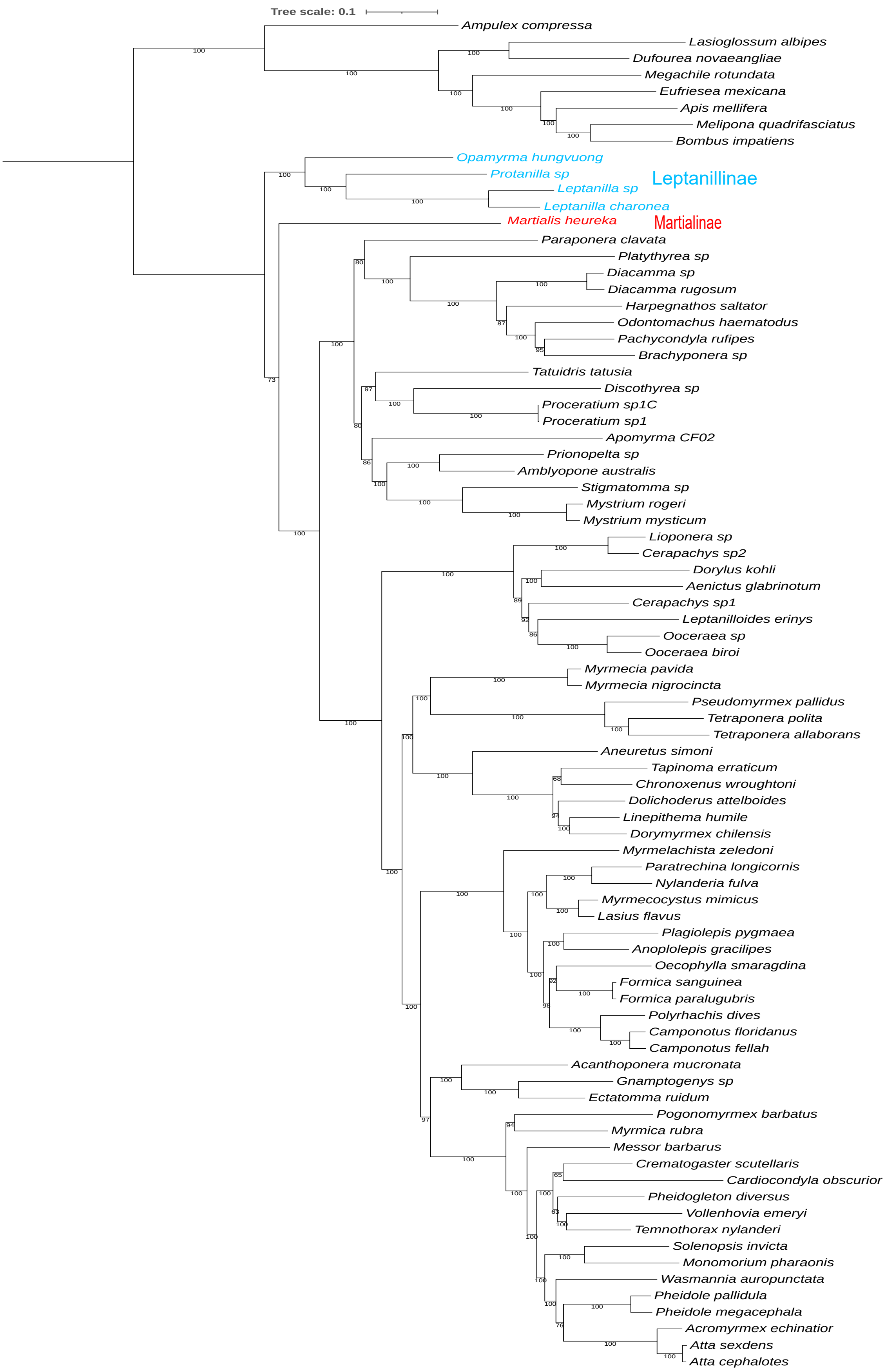

Figure S6. Phylogenomic analyses of trimmed nuclear genomic 4,151-gene dataset (Matrix 5) from Romiguier et al. (2022). Under the heterotachous GHOST (LG+FO*H4) model in IQ-TREE.
