# Supplementary material for: Ant backbone phylogeny resolved by modelling compositional heterogeneity among sites in genomic data
Source: Commun Biol. 2024 Jan 17;7:106. doi: 10.1038/s42003-024-05793-7 (PMC10794244; doi:10.1038/s42003-024-05793-7)
Supplement: Supplementary file 2 — Reporting Summary [file 42003_2024_5793_MOESM2_ESM.pdf]

Corresponding author(s): COMMSBIO-23-2254A

Last updated by author(s): Oct 11, 2023

# Reporting Summary

Nature Portfolio wishes to improve the reproducibility of the work that we publish. This form provides structure for consistency and transparency in reporting. For further information on Nature Portfolio policies, see our Editorial Policies and the Editorial Policy Checklist.

## Statistics

For all statistical analyses, confirm that the following items are present in the figure legend, table legend, main text, or Methods section.

| n/a | Confirmed | |
|---|---|---|
| ☒ | ☐ | The exact sample size (*n*) for each experimental group/condition, given as a discrete number and unit of measurement |
| ☒ | ☐ | A statement on whether measurements were taken from distinct samples or whether the same sample was measured repeatedly |
| ☒ | ☐ | The statistical test(s) used AND whether they are one- or two-sided<br>*Only common tests should be described solely by name; describe more complex techniques in the Methods section.* |
| ☒ | ☐ | A description of all covariates tested |
| ☒ | ☐ | A description of any assumptions or corrections, such as tests of normality and adjustment for multiple comparisons |
| ☒ | ☐ | A full description of the statistical parameters including central tendency (e.g. means) or other basic estimates (e.g. regression coefficient) AND variation (e.g. standard deviation) or associated estimates of uncertainty (e.g. confidence intervals) |
| ☒ | ☐ | For null hypothesis testing, the test statistic (e.g. *F*, *t*, *r*) with confidence intervals, effect sizes, degrees of freedom and *P* value noted<br>*Give P values as exact values whenever suitable.* |
| ☐ | ☒ | For Bayesian analysis, information on the choice of priors and Markov chain Monte Carlo settings |
| ☒ | ☐ | For hierarchical and complex designs, identification of the appropriate level for tests and full reporting of outcomes |
| ☒ | ☐ | Estimates of effect sizes (e.g. Cohen's *d*, Pearson's *r*), indicating how they were calculated |

*Our web collection on statistics for biologists contains articles on many of the points above.*

## Software and code

Policy information about availability of computer code

**Data collection**

I used the most comprehensive Sanger-sequencing and nuclear genome alignments from Borowiec et al. 2019 and Romiguier et al. 2022, respectively. The datasets were downloaded from the Zenodo data repository (Sanger-sequencing datasets: https://doi.org/10.5281/zenodo.2549806; genome-scale datasets: https://doi.org/10.5281/zenodo.5705739).
For the Sanger-sequencing (11 nuclear loci) data, I used all four nucleotide [NT] matrices generated in Borowiec et al. 2019: 1) Full 11-locus matrix (123 taxa, 7,451 NT sites); 2) Full matrix with the most AT-rich outgroups excluded (117 taxa, 7,451 NT sites); 3) Full matrix with the most GC-rich outgroups excluded (117 taxa, 7,451 NT sites); and 4) Homogeneous matrix with heterogeneous partitions removed (123 taxa, 3,995 NT sites).
For the nuclear genomic data, I used the two BUSCO-gene amino acid [AA] supermatrices from Romiguier et al. 2022: 1) Fewer-outgroup AA dataset (83 taxa, 4,151 single-copy protein-coding genes, 1,692,050 AA sites); and 2) More-outgroup amino acid dataset (188 taxa, 2,343 single-copy protein-coding genes, 983,951 AA sites), which was designed to test the impact of outgroup selection on tree inference. To balance taxon sampling of subfamilies, focus on deeper phylogeny of ants, and more importantly, speed up computationally heavy Bayesian runs, I subsampled the 4,151-gene supermatrix and filtered constant sites using BMGE v.1.1 [21], resulting in Matrix 1 (38 taxa, 647,114 AA sites). Similarly, I randomly pruned the outgroup taxa and selected all representative ingroup genera of the 2,343-gene supermatrix, and filtered ambiguously aligned sites using BMGE with default setting (-m BLOSUM62, -h 0.5), yielding Matrix 2 (47 taxa, 623,908 AA sites).
Additionally, as sensitivity tests of the potential impact of my data filtering and subsampling methods on tree inference, I 1) filtered the 2,343-gene supermatrix using a stringent setting (-m BLOSUM30, -h 0.1:0.5) to select slow-evolving AA sites, resulting in Matrix 3 (47 taxa, 95,201 AA sites); 2) removed remotely related outgroup and selected representative ant genera, but kept all AA sites of the 4,151-gene supermatrix, resulting in Matrix 4 (17 taxa, 1,692,050 AA sites); and 3) filtered the 4,151-gene supermatrix using BMGE with a very stringent setting (-m BLOSUM30 -h 0.2:0.3), resulting in Matrix 5 (82 taxa, 21,902 AA sites).

**Data analysis**

(1) Phylogenetic analyses

| | |
|---|---|
| Data analysis | Phylogenomic analyses of the nuclear genomic datasets, Matrices 1–5, were conducted using the simpler LG4X+R model [22] and the site-heterogeneous model (LG+C20+F+G) [23] with IQ-TREE v.2.1.3 [24]. For the site-heterogeneous LG+C20+F+G model, the posterior mean site frequency (PMSF) model [25] was applied using the respective LG4X+R tree as the guide tree. In addition, the comparatively small Matrix 3 was analyzed using the site-homogeneous model (LG+F+G) and the site-heterogeneous models (LG+C40+F+G, and LG+C60+F+G) with IQ-TREE v.2.1.3, corresponding to the models used on the following model comparison (see below).<br>As the subfamilial interrelationships of ants are expected to be affected by long-branch attraction artefacts [1,2], I used the compositionally site-heterogeneous infinite mixture model CAT-GTR+G4 implemented in PhyloBayes MPI 1.9 [26] which has been proven to be effective for mitigating such a systematic error by modelling across-site compositional heterogeneity. Four Sanger-sequencing matrices (as nucleotide) and five genome-based supermatrices (as amino acid) were analyzed under the CAT-GTR+G4 model. For each analysis two Markov chain Monte Carlo chains were run and convergence was assessed using the bpcomp and tracecomp tools implemented in PhyloBayes [27]. Approximately 30% of samples were discarded as burn-in. Detailed information about the PhyloBayes runs (burnin samples, total number of cycles, bpcomp maxdiff, and tracecomp minimal overall effective size) was given in the figure caption of each analysis.<br><br>(2) Model comparison<br>For the filtered genome-scale AA dataset (Matrix 3), we used the comparatively efficient and reliable approaches, i.e, the leave-one-out cross-validation (LOO-CV) and the widely applicable information criterion (wAIC) [28], to estimate the relative fit of alternative models (CAT-GTR, LG+G, LG+C20, LG+C40, and LG+C60) in the latest PhyloBayes MPI 1.9. The LOO-CV and wAIC scores were compared to determine and select the best-fitting model, based on which my preferred tree of ants was selected.<br><br>(3) Testing model adequacy<br>Posterior Predictive Analyses (PPA) were performed on Matrix 3 using PhyloBayes MPI 1.9 to test whether LG+F+G, LG+C20+F+G, LG+C40+F+G, LG+C60+F+G, or CAT-GTR+G can adequately describe site-specific amino acid preferences for the dataset. These models (especially LG+F+G and LG+C20+F+G) were selected and tested because they had previously been used in the recent study of ant phylogenomics [2] that yielded a contradictory topology to my preferred tree (figure 2a). |

For manuscripts utilizing custom algorithms or software that are central to the research but not yet described in published literature, software must be made available to editors and reviewers. We strongly encourage code deposition in a community repository (e.g. GitHub). See the Nature Portfolio guidelines for submitting code & software for further information.

## Data

Policy information about availability of data

All manuscripts must include a data availability statement. This statement should provide the following information, where applicable:

- Accession codes, unique identifiers, or web links for publicly available datasets
- A description of any restrictions on data availability
- For clinical datasets or third party data, please ensure that the statement adheres to our policy

All data sets and output files are available from DRYAD, https://doi.org/10.5061/dryad.fxpnvx0w7
Also from this link: https://datadryad.org/stash/share/xSma8zOHnHjLNinEI7A1jv5B-i7erScJDLjBEGH1T80

## Research involving human participants, their data, or biological material

Policy information about studies with human participants or human data. See also policy information about sex, gender (identity/presentation), and sexual orientation and race, ethnicity and racism.

| | |
|---|---|
| Reporting on sex and gender | n/a |
| Reporting on race, ethnicity, or other socially relevant groupings | n/a |
| Population characteristics | n/a |
| Recruitment | n/a |
| Ethics oversight | n/a |

Note that full information on the approval of the study protocol must also be provided in the manuscript.

# Field-specific reporting

Please select the one below that is the best fit for your research. If you are not sure, read the appropriate sections before making your selection.

☒ Life sciences ☐ Behavioural & social sciences ☐ Ecological, evolutionary & environmental sciences

For a reference copy of the document with all sections, see nature.com/documents/nr-reporting-summary-flat.pdf

# Life sciences study design

All studies must disclose on these points even when the disclosure is negative.

| | |
|---|---|
| Sample size | n/a |
| Data exclusions | n/a |
| Replication | n/a |
| Randomization | n/a |
| Blinding | n/a |

# Reporting for specific materials, systems and methods

We require information from authors about some types of materials, experimental systems and methods used in many studies. Here, indicate whether each material, system or method listed is relevant to your study. If you are not sure if a list item applies to your research, read the appropriate section before selecting a response.

## Materials & experimental systems

| n/a | Involved in the study | |
|---|---|---|
| ☒ | ☐ | Antibodies |
| ☒ | ☐ | Eukaryotic cell lines |
| ☒ | ☐ | Palaeontology and archaeology |
| ☐ | ☒ | Animals and other organisms |
| ☒ | ☐ | Clinical data |
| ☒ | ☐ | Dual use research of concern |
| ☒ | ☐ | Plants |

## Methods

| n/a | Involved in the study | |
|---|---|---|
| ☒ | ☐ | ChIP-seq |
| ☒ | ☐ | Flow cytometry |
| ☒ | ☐ | MRI-based neuroimaging |

## Animals and other research organisms

Policy information about studies involving animals; ARRIVE guidelines recommended for reporting animal research, and Sex and Gender in Research

| | |
|---|---|
| Laboratory animals | Ants species sampled in two previous publications: Borowiec et al. 2019 and Romiguier et al. 2022. No new extant species were sampled in our study. |
| Wild animals | No new extant species were sampled in our study. |
| Reporting on sex | No new extant species were sampled in our study. |
| Field-collected samples | No new extant species were sampled in our study. |
| Ethics oversight | Ants species sampled in two previous publications: Borowiec et al. 2019 and Romiguier et al. 2022. No new extant species added. |

Note that full information on the approval of the study protocol must also be provided in the manuscript.
